# Supplementary material for: Multiple axes of visual system diversity in Ithomiini, an ecologically diverse tribe of mimetic butterflies
Source: J Exp Biol. 2023 Dec 8;226(24):jeb246423. doi: 10.1242/jeb.246423 (PMC10714147; doi:10.1242/jeb.246423)
Supplement: Supplementary information [file jexbio-226-246423-s1.pdf]

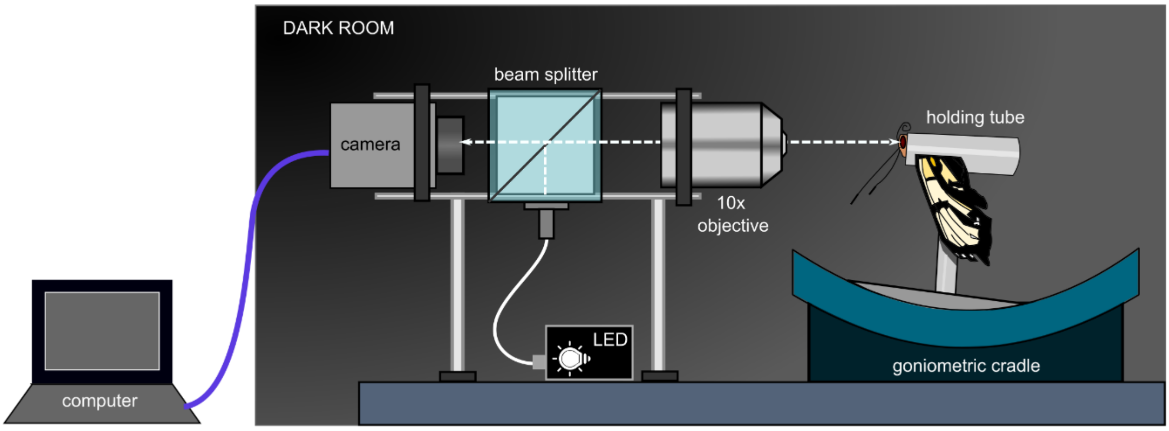

Fig. S1. Ophthalmoscope apparatus used to image the eyeshine of live butterflies after dark adaptation. An LED light source provided coaxial illumination to the compound eye via a beam splitter and 10x objective lens. Butterflies were mounted in holding tubes, immobilised using plasticine, and suspended at the centre of rotation of a goniometric cradle. A camera connected to a laptop with the uEye Cockpit program installed was used to record eyeshine images.

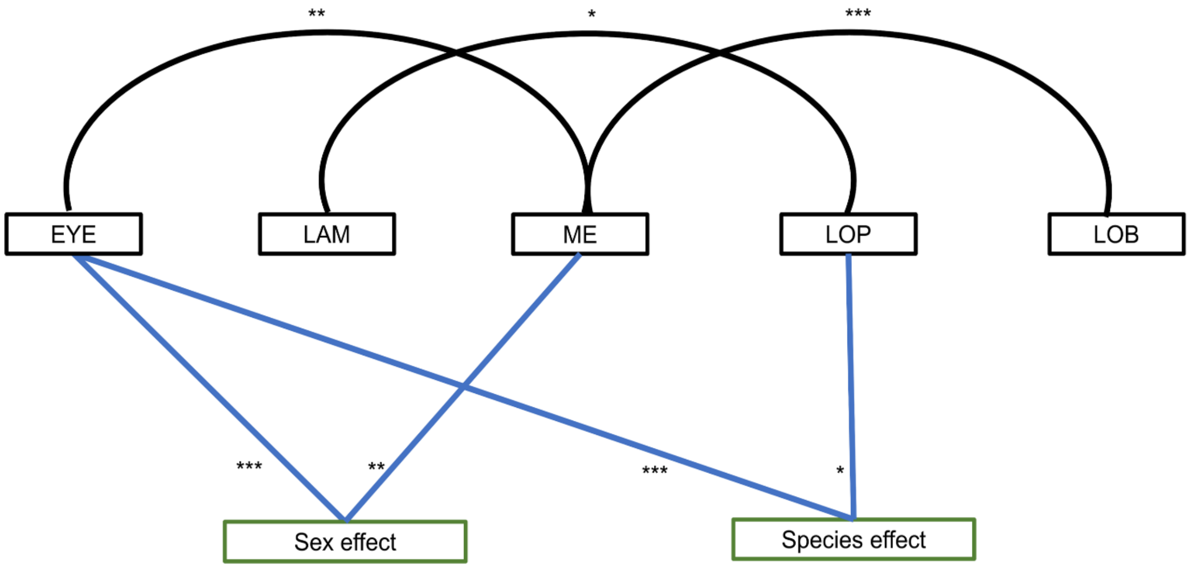

Fig. S2. Patterns of volumetric covariance between the four main optic lobe neuropils and the eye (EYE). Significant covariance is shown by solid black lines and structures which show significant sex/species effects after controlling for covariance are indicated by solid blue lines. NS $P > 0.05$, *$P < 0.05$, **$P < 0.01$, ***$P < 0.001$.

Journal of Experimental Biology • Supplementary information

Table S1. Opsin sequences

Available for download at
https://journals.biologists.com/jeb/article-lookup/doi/10.1242/jeb.246423#supplementary-data

Table S2. Eyeshine raw data

Available for download at
https://journals.biologists.com/jeb/article-lookup/doi/10.1242/jeb.246423#supplementary-data

Table S3. Anatomical raw data

Available for download at
https://journals.biologists.com/jeb/article-lookup/doi/10.1242/jeb.246423#supplementary-data

Table S4. Eyeshine analysis

Available for download at
https://journals.biologists.com/jeb/article-lookup/doi/10.1242/jeb.246423#supplementary-data

Table S5. Micro-CT parameters

Available for download at
https://journals.biologists.com/jeb/article-lookup/doi/10.1242/jeb.246423#supplementary-data

Table S6. Micro-CT eye and neuronanatomical comparisons

Available for download at
https://journals.biologists.com/jeb/article-lookup/doi/10.1242/jeb.246423#supplementary-data
